# Reply to the Letter to the Editor: Blind spots in radiology leadership regarding human resources and organization management in the era of artificial intelligence

**DOI:** 10.1007/s00330-026-12422-w

**Published:** 2026-02-27

**Authors:** Barbara D. Wichtmann, Daniel Paech, Oleg S. Pianykh, Susie Y. Huang, Steven E. Seltzer, James Brink, Fiona M. Fennessy

**Affiliations:** 1 Clinic of Neuroradiology, University Hospital Bonn, Bonn, Germany; 2 Department of Radiology, Mass General Brigham, Boston, MA USA; 3 German Center for Neurodegenerative Diseases (DZNE), Bonn, Germany

Dear Editor,

We thank Hongnan Ye for his thoughtful and constructive commentary on our recent article, “Leadership in radiology in the era of technological advancements and artificial intelligence” [1], highlighting our discussion of the critical role of leadership and governance in ensuring the ethical, effective, and patient-centered integration of AI to optimize workflows, enhance diagnostic efficiency, and advance precision medicine. We appreciate the opportunity to further expand on the human resource and organizational implications of AI adoption in radiology, including its effects on professional autonomy, performance evaluation, and leadership responsibilities.

The letter appropriately draws attention to cognitive offloading as a potential risk inherent to any assistive technology designed to augment human performance [2]. As a similar example, one might caution against the deployment of robotic surgical systems because surgeons may lose their highly refined human surgical skills.

While cognitive offloading is a genuine phenomenon that warrants attention, two factors in particular should not deter the responsible exploration and deployment of AI.

First, although technological innovation inevitably entails trade-offs, leadership decisions should be guided by the overarching goal of improving patient care.

Second, experience from the long-standing clinical use of computer-aided diagnosis (CAD) in breast imaging suggests that cognitive offloading is not an inevitable consequence of such technologies. FDA-approved mammography CAD systems have been used in routine practice for more than two decades to direct attention to regions of interest, while interpretation and final diagnostic responsibility remain with the radiologist [3]. Evidence from mammography CAD and population-based AI screening trials shows that radiologists do not blindly follow algorithmic outputs, as reflected in a tendency to underweight AI flags in screening consensus decisions [4, 5]. In fact, under-reliance on AI rather than uncritical acceptance may currently limit its potential benefit [6]. Still, automation bias is a well-described phenomenon in human–AI interaction, with experimental mammography studies showing that AI suggestions can influence reader decisions, particularly among less experienced readers [7]. These observations argue not against the use of assistive technologies, but for training, supervision, and audit structures that foster calibrated trust in AI. Leadership should therefore prioritize education and governance over avoidance, ensuring that AI augments expertise while preserving accountability [8].

Looking ahead, Hongnan Ye also raises an important management challenge that radiology leaders will increasingly face as AI becomes embedded in routine clinical production. As AI assistance elevates baseline performance across a department, traditional output-focused metrics such as report volume, turnaround time, or even accuracy may lose discriminatory power. In this setting, excellence is less likely to be defined by what is produced than by how it is produced. We agree that this shift creates an opportunity to rethink performance assessment toward process-oriented indicators that capture uniquely human contributions within AI-supported workflows, including clinical judgment in ambiguous cases, critical appraisal of algorithmic outputs, interdisciplinary communication, and stewardship of quality and safety [8]. Developing and implementing such metrics will require leaders to be both technologically literate and organizationally adept, with sufficient understanding of AI capabilities and limitations to ensure that evaluation systems continue to reward expertise, insight, and accountability rather than mere proficiency with tools [9].

Lastly, the letter envisions a new type of workplace in which AI systems will assume increasingly autonomous operational roles within radiology workflows. As AI moves beyond decision support toward performing bounded tasks such as worklist triage [10], protocol optimization [11], or draft report generation and structured data extraction [12, 13], radiology leaders will be required to manage not only human professionals but hybrid human–AI teams. In this setting, leadership responsibilities shift from the question of whether AI should be used to how its capabilities can be identified, governed, and deployed in a rational and clinically meaningful manner to improve system performance and patient care [14, 15].

Importantly, this challenge is not without precedent. Radiology has repeatedly demonstrated a strong capacity to integrate disruptive technologies into routine clinical practice. A prominent example is the transition from analog film-based workflows to fully digital imaging ecosystems, catalyzed by the introduction of the ACR–NEMA digital imaging standards in the mid-1980s and their subsequent evolution into the DICOM standard, which enabled interoperable, network-based image exchange across vendors and institutions [16, 17]. The introduction of picture archiving and communication systems, network-enabled image distribution that laid the technical foundation for teleradiology, and enterprise-wide image distribution required substantial redesign of workflows, redefinition of professional roles, and the establishment of formal standards and conformance mechanisms to ensure interoperability, quality, safety, and accountability. The resulting paradigm, enabling access to any image anywhere and anytime, would have been difficult to anticipate only a few decades ago and was realized through sustained leadership, interdisciplinary collaboration, and organizational adaptability. Compared with other diagnostic disciplines, like anatomic pathology, that have undergone slower or more fragmented digital transitions, radiology’s experience suggests that our specialty is well-positioned to navigate the organizational implications of increasingly autonomous AI systems, provided their deployment is guided by robust governance, continuous evaluation, and clear human oversight.

In closing, we appreciate Hongnan Ye’s cogent arguments and thoughtful suggestions. We are confident that radiology leaders will be able to optimize the deployment of AI systems to improve diagnostic performance while avoiding many of the potential unintended consequences of this technological transformation. Doing so, however, will require deliberate preparation, informed leadership, and sustained organizational commitment.
